# Molecular Docking of Key Compounds from Acacia Honey and *Nigella sativa* Oil and Experimental Validation for Colitis Treatment in Albino Mice

**DOI:** 10.3390/biology13121035

**Published:** 2024-12-11

**Authors:** Mehwish Bibi, Irfan Baboo, Hamid Majeed, Santosh Kumar, Maximilian Lackner

**Affiliations:** 1 Department of Zoology, Cholistan University of Veterinary and Animal Sciences (CUVAS), Bahawalpur 63100, Pakistan; mehwishmanzoor@gmail.com (M.B.); santoshkumar@cuvas.edu.pk (S.K.); 2 Department of Food Science and Technology, Cholistan University of Veterinary and Animal Sciences (CUVAS), Bahawalpur 63100, Pakistan; hamidmajeed@cuvas.edu.pk; 3 Department of Industrial Engineering, University of Applied Sciences Technikum Wien, 17 Hoechstaedtplatz 6, 1200 Vienna, Austria

**Keywords:** *Nigella sativa*, acacia honey, albino mice, oil, colitis

## Abstract

Colitis, an inflammatory condition of the colon that encompasses ulcerative colitis (UC) and Crohn’s disease, presents significant challenges due to the limitations and side effects of current treatments. This study investigates the potential of natural products, specifically AH and NSO, as organic therapeutic agents for colitis. Molecular docking studies were conducted to identify the binding affinities and interaction mechanisms between the bioactive compounds in AH and NSO and proteins implicated in colitis, such as those involved in inflammation and oxidative stress pathways. An in vivo experiment was performed using an albino mouse model of colitis, with clinical symptoms, histopathological assessments, and biochemical analyses conducted to evaluate the therapeutic effects of the compounds both individually and in combination. Results from the molecular docking studies revealed promising binding interactions between fructose and Prostaglandin G/H synthase 2 (Ptgs2) and between fructose and cellular tumor antigen p53, with docking energy measured at −6.0 kcal/mol and −5.1 kcal/mol, respectively. Meanwhile, the presence of glucose molecule glucokinase chain A (−6.3 kcal/mol) and chain B (−5.8 kcal/mol) indicated potential efficacy in modulating inflammatory pathways. Experimental data demonstrated that treatment with AH and NSO significantly reduced inflammation, improved gut health, and ameliorated colitis symptoms. Histopathological evaluations confirmed reduced mucosal damage and immune cell infiltration, while biochemical analyses showed normalization of inflammatory markers and oxidative stress levels. This study provides compelling evidence for the potential of AH and NSO as natural, complementary treatments for colitis, suggesting their future role in integrative therapeutic strategies. However, further research into long-term safety, optimal dosing, and mechanisms of action is warranted to translate these findings into clinical applications.

## Simple Summary

This study investigated the use of natural remedies, acacia honey (AH) and *Nigella sativa* oil (NSO), to treat colitis, a painful inflammation of the colon. Current treatments often have side effects, leading researchers to explore safer alternatives. The study used computer simulations to understand how these natural products interact with proteins linked to inflammation and oxidative stress, key factors in colitis. Experiments on mice with colitis revealed that both AH and NSO significantly reduced inflammation, restored colon health, and improved symptoms, with a combination of the two showing the greatest healing effects. The treatments were well-tolerated, with no negative impact on the mice’s overall health. These findings suggest that AH and NSO could provide a natural, effective, and safe approach to managing colitis, offering hope for improved care and quality of life for those affected.

## 1. Introduction

Colitis, an inflammatory condition of the colon, manifests through symptoms such as abdominal pain, diarrhea, rectal bleeding, and weight loss [1]. It encompasses both UC and Crohn’s disease, collectively known as inflammatory bowel disease (IBD) [2]. Although the exact cause of IBD remains uncertain, it is generally believed to result from a combination of genetic predisposition, environmental influences, gut microbiota imbalance, and immune system dysfunction [3]. Current treatments, such as corticosteroids, immunosuppressants, and biologics, can alleviate symptoms but often carry significant side effects and risks of long-term complications [4]. This highlights the need for alternative, natural, and safer treatment options to enhance or replace conventional approaches.

Recently, the therapeutic potential of natural products has gained attention, especially those derived from plants and traditional remedies [5]. Among these, AH and NSO have shown promise for their anti-inflammatory and antioxidant properties [6]. Both have been used in traditional medicine for centuries for a variety of ailments, including digestive and inflammatory disorders [7]. Modern studies indicate that the active compounds in AH and NSO could help reduce inflammation, combat oxidative stress, and improve gut health, suggesting potential benefits in managing colitis [8,9].

AH is distinguished by its high content of phenolic acids, flavonoids, and hydrogen peroxide, which endow it with significant anti-inflammatory, antioxidant, and antimicrobial properties [10]. Studies have demonstrated that AH can modulate immune responses and inhibit pro-inflammatory enzymes, making it an effective candidate for addressing inflammatory conditions like colitis [11]. The antioxidant properties of AH help neutralize free radicals and reduce oxidative stress in the colon, which is a key contributor to tissue damage and inflammation in colitis [12]. Experimental evidence has shown that AH can improve gut barrier function, enhance mucosal healing, and decrease inflammation in animal models of colitis [13].

NSO is extracted from black seeds and is well-known for its broad-spectrum pharmacological properties, including anti-inflammatory, antioxidant, and immunomodulatory effects [14]. Its most studied active component, thymoquinone, has been shown to regulate immune cell activity, reduce pro-inflammatory cytokine production, and inhibit pathways such as NF-κB, which play a central role in inflammatory responses [15]. NSO has the potential to combat oxidative stress, protect epithelial cells, and reduce inflammation, which makes it a valuable candidate for colitis treatment [16]. Research has highlighted the effectiveness of NSO in reducing colitis symptoms, improving histological features, and normalizing serum inflammation markers [17].

The use of molecular docking technology provides a powerful tool for understanding how bioactive compounds interact with proteins involved in disease [18]. This computational method simulates the binding of compounds to target proteins, predicting their binding affinity and potential effectiveness [19]. Molecular docking helps identify the most promising compounds for further investigation, streamlining the selection process before moving to experimental testing [20]. By modeling the interactions between bioactive molecules and proteins, researchers can gain insight into the potential mechanisms of action of these compounds [21].

While molecular docking provides valuable predictive data, experimental verification by in vivo studies is crucial for confirming the biological activity of these compounds [22]. In vivo studies using animal models, such as albino mice, offer a means of evaluating the real-world effects of AH and NSO. In these studies, researchers can assess clinical symptoms, histopathological changes, and biochemical markers to determine the effectiveness of the treatment [23]. Mice are particularly advantageous for such studies due to their genetic uniformity, ease of monitoring, and well-established response to experimental colitis models [24]. These studies provide a comprehensive evaluation of how bioactive compounds impact inflammation, oxidative stress, and gut health.

Despite promising preliminary studies, to understand the potential of AH and NSO for colitis treatment, there is a need for further investigation, including elucidating the molecular mechanisms by which these substances reduce inflammation, comparing their individual and combined effects, and determining optimal dosing and formulation strategies. Their long-term safety and potential toxicity, as well as interactions with other medications, also need to be explored. Current in vivo studies often rely on limited models, emphasizing the need for more-diverse and long-term research. Additionally, molecular docking predictions are useful for identifying promising compounds to treat infections. To fulfill this research gap, the present study was designed to investigate the therapeutic potential of AH and NSO for colitis treatment.

## 2. Materials and Methods

### 2.1. Active Compound Screening

After a recent literature survey of PubMed and Google Scholar, it was observed that thymoquinone is the major active compound of NSO [25,26], while glucose and fructose are those of AH [27,28].

### 2.2. Drug Target Prediction

The Stitch (http://stitch.embl.de/, accessed on 20 May 2024) database was used to identify drug target prediction on 20 May 2024 by adding “thymoquinone”, “glucose”, and “fructose” to the item name search box, while “Mus Musculus” was selected as the organism name. Proteins with maximum similarity scores were shortlisted for further investigation. Validation of the selected protein structures was carried out using PROCHECK (https://www.ebi.ac.uk/thornton-srv/software/PROCHECK, accessed on 20 May 2024) through the generation of a Ramachandran plot [29].

### 2.3. Molecular Docking

The chemical structures of “thymoquinone” “glucose”, and “fructose” were obtained from the PubChem database (https://pubchem.ncbi.nlm.nih.gov/ accessed on 25 May 2024). It was observed that chemical databases have different orientations of fructose, so we downloaded beta-D-fructose (PubChem CID 10267), glucose (PubChem CID 5793), and thymoquinone (PubChem CID 10281) in “sdf” format for further analysis. The 3D structures of interacting proteins were retrieved in the “pdb” format from the Protein Data Bank (https://www.rcsb.org/ accessed on 28 May 2024). Molecular docking studies were performed using SwissDock (https://www.swissdock.ch/, accessed on 20 May 2024) from the Swiss Institute of Bioinformatics on 1st June 2024. Ligands exhibiting the highest binding free energy were considered to have the most favorable interactions with the receptor. The docking results were visualized in 3D, and solid-surface representations were created using UCSF Chimera version 1.17.3. Additionally, 2D interaction diagrams were generated using BIOVIA Discovery Studio Visualizer (version 4.5), providing detailed insight into binding interactions [30].

### 2.4. Sample Collection

AH samples were collected in August 2023 during the flowering season from the district Bahawalpur, Pakistan. Pure AH samples were directly collected from sources in small, sterilized glass bottles. For each honey sample, different concentrations (2%, 4%, 6%, and 8%/v) were prepared. Pure NSO was purchased from the market Karachi Essence Bahawalpur, a certified vendor of Sigma Aldrich in Pakistan.

### 2.5. Protective Effect of AH and Nigella sativa Oil Against Colitis

#### 2.5.1. Experimental Animals

Young male Wistar albino mice (19–24 g, eight weeks old) were obtained from the Department of Physiology, the Islamia University Bahawalpur, Pakistan. The mice were placed in polypropylene cages and maintained on a standard laboratory diet and free access to water under a normal light/dark cycle. The mice were allowed to acclimatize for one week prior to the commencement of experiments. The animals were divided into five groups, each with ten animals. The negative control (NC) received no treatment and induction of colitis. The positive control (PC) was the colitis-induced group that received no treatment. The NSO group received 20 µL of pure NSO. The AH group orally received 1 mL of natural unprocessed honey. The AH + NSO group received 1 mL of AH and 20 µL of NSO.

#### 2.5.2. Induction of Colitis

Colitis was induced by oral administration of *Salmonella typhimurium* at a dosage of 10^4^ CFU via oral gavage to all experimental groups except the control group. The tested treatments of AH (1 mL), NSO (20 µL), and AH (1 mL) + NSO (20 µL) were administered orally twice a day for 15 days. Colitis symptoms were confirmed in experimental groups on the fourth day by the identification of nonstick, loose stool. Morphological assessment was undertaken to examine the clinical activity of colitis. Throughout the experimental period, stool consistency, individual animal weight, and fecal blood were evaluated and scored accordingly. Total clinical scores (TCSs) were calculated according to Rezaei et al. [31]. This involved summing the scores of three parameters (body weight loss, bleeding, and stool consistency) and dividing the result by 3. The scoring criteria were as follows: body weight loss (0: none, 1: 1–5%, 2: 6–10%, 3: >11%), bleeding (0: negative, 1: slight bleeding, 2: moderate bleeding, 3: severe bleeding), and stool consistency (0: regular, 1: soft unformed excrement, 2: loose stool, 3: watery diarrhea). On the 15th day, all animals from each experimental group were anesthetized properly and then slaughtered to obtain the distal portion of the colon for histological and biochemical analysis.

#### 2.5.3. Measurement of Oxidative Stress and Enzymatic Activity Markers

To analyze the colon tissue supernatants for protein content and the activities of TBARS, superoxide dismutase (SOD), catalase (CAT), and myeloperoxidase (MPO), a standardized protocol was followed. Colon tissue samples were collected and washed with cold phosphate-buffered saline (PBS) to remove any blood or contaminants. The tissues were then cut into small pieces and placed into a homogenization tube, where they were mixed with a homogenization buffer containing a protease inhibitor cocktail. The homogenization process was conducted using a tissue homogenizer until a uniform suspension was obtained. The homogenate was then transferred to a microcentrifuge tube and centrifuged at 12,000× *g* for 15–20 min at 4 °C to separate the soluble proteins from the debris. The supernatant, which contained soluble proteins, was collected and stored at −80 °C for subsequent analysis.

##### Protein Quantification Using the BCA Assay

The protein content in the supernatant was quantified using a BCA Assay Kit (Servicebio Co., Ltd., Wuhan, China; Cat #G2026-1000 T). To perform the assay, a reagent mix was prepared by combining reagent A and reagent B in a 50:1 ratio, as per the manufacturer’s guidelines. A 20 µL volume of the colon tissue supernatant was added to each well of a 96-well plate, followed by the addition of 200 µL of the prepared BCA reagent mix. The plate was incubated at 37 °C for 30 min, and the absorbance was read at 562 nm using a microplate reader. The protein concentration in the samples was calculated by plotting the standard curve and determining the corresponding values [32].

##### TBARS (Thiobarbituric Acid Reactive Substances) Assay

To measure the TBARS activity, the supernatant was mixed with trichloroacetic acid (TCA) and thiobarbituric acid (TBA) reagent. Specifically, 100 µL of the supernatant was combined with 200 µL of TCA and 100 µL of the TBA reagent. The reaction mixture was incubated at 95 °C for 60 min to facilitate the reaction. After incubation, the samples were cooled and centrifuged at 12,000× *g* for 10 min to separate the precipitate. The supernatant was transferred to a cuvette, and the absorbance was measured at 532 nm using a spectrophotometer to determine the TBARS content [33].

##### SOD (Superoxide Dismutase) Activity Assay

The SOD activity was assessed using an assay that involved a reaction mixture prepared with assay buffer, xanthine, xanthine oxidase, and nitroblue tetrazolium (NBT). Fifty microliters of the colon tissue supernatant were added to 950 µL of assay buffer, and the mixture was incubated at 37 °C for 20 min. After incubation, 50 µL of the NBT reagent was added to each well, and the absorbance was measured at 560 nm to quantify the SOD activity [34].

##### CAT (Catalase) Activity Assay

Catalase activity was measured by preparing a reaction mixture containing 100 µL of the colon tissue supernatant and 900 µL of 0.1 M phosphate buffer (pH 7.0). To initiate the reaction, 1 mL of 10 mM hydrogen peroxide (H_2_O_2_) solution was added to the mixture, and the absorbance at 240 nm was measured over a 1–2 min period. This process helped determine the catalase activity based on the rate of H_2_O_2_ decomposition [35].

##### MPO (Myeloperoxidase) Activity Assay

MPO activity was determined by mixing 50 µL of the colon tissue supernatant with 950 µL of phosphate buffer (pH 6.0) containing o-dianisidine. Following this, 50 µL of a 3% hydrogen peroxide solution was added, and the reaction was incubated at room temperature for 5 min. The absorbance was measured at 460 nm using a spectrophotometer to quantify the MPO activity [36].

#### 2.5.4. Histopathological Study

Following necropsy, tissue samples from the colon were fixed in 4% paraformaldehyde, embedded in paraffin, and sectioned into 5 μm slices. Hematoxylin and eosin (H&E) staining was performed on these sections, which were then examined under an Olympus BX53 microscope (Olympus, Tokyo, Japan) with ×400 magnification power. Images were acquired using Olympus cellSens Entry 1.14 software (Olympus).

### 2.6. Statistical Analysis

The statistical software SPSS (version 22) was used for analyzing the data, and Origin (version 8.6) was deployed for graph preparation. Results were presented as mean ± the standard error of the mean (SEM). Statistical analysis involved repeated measures ANOVA for body weight loss and one-way ANOVA followed by Tukey’s multiple comparison test for other variables. A *p*-value of less than 0.05 was considered statistically significant.

## 3. Results

### 3.1. Ligand–Protein Interaction

**Figure 1:**
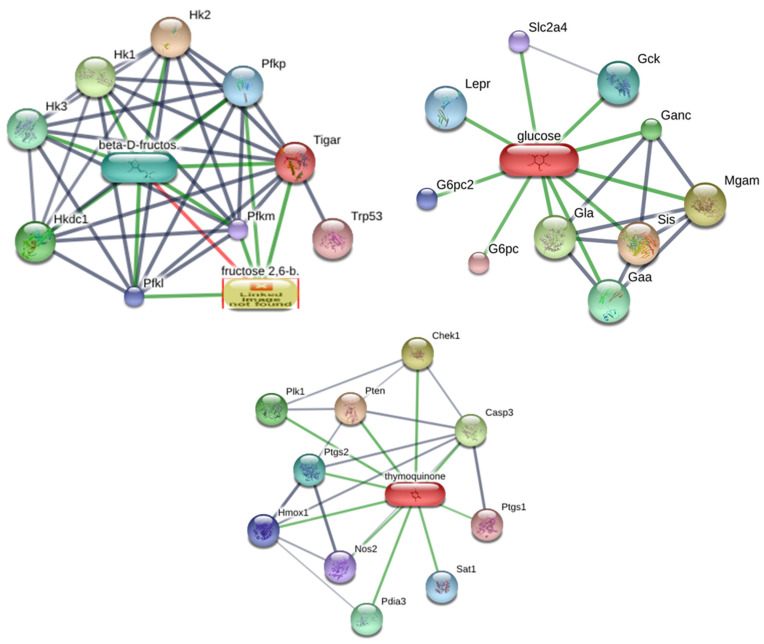
Ligand–protein and protein–protein interaction.

Beta-D-fructose is connected to multiple proteins such as HK1, HK2, HK3, Hkdc1, Pfkp, Pfkl, Pfkfb, Tigar, and Pfkf, while thymoquinone interacts with Plk1, Pten, Chek1, Casp3, Ptgs1, Ptgs2, Hmox1, Nos2, Sat1, Pdia3, and Trp53. The connections are made via colored edges: Green lines indicate positive regulatory interactions. Blue lines indicate protein–protein interaction. Red lines (e.g., leading to “fructose 2,6-b”) indicate a missing link (Figure 1).

### 3.2. Molecular Docking Analysis

**Figure 2:**
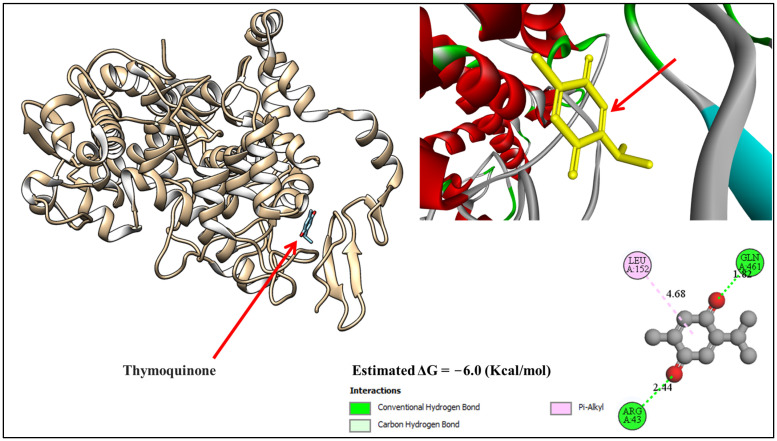
Docking results of Thymoquinone with Prostaglandin G/H synthase 2 (PDB: 4FM5).

The molecular docking interaction between thymoquinone and Prostaglandin G/H synthase 2 occurred with a docking energy level of −6.0 kcal/mol. Detailed annotations highlight key binding interactions, including conventional hydrogen bonds between thymoquinone and Arginine 43 and Glutamine 461 with distances of 2.44 Å and 1.82 Å, as well as interactions between Pi-alkyl and leucine with a distance of 4.68 Å. (Figure 2).

**Figure 3:**
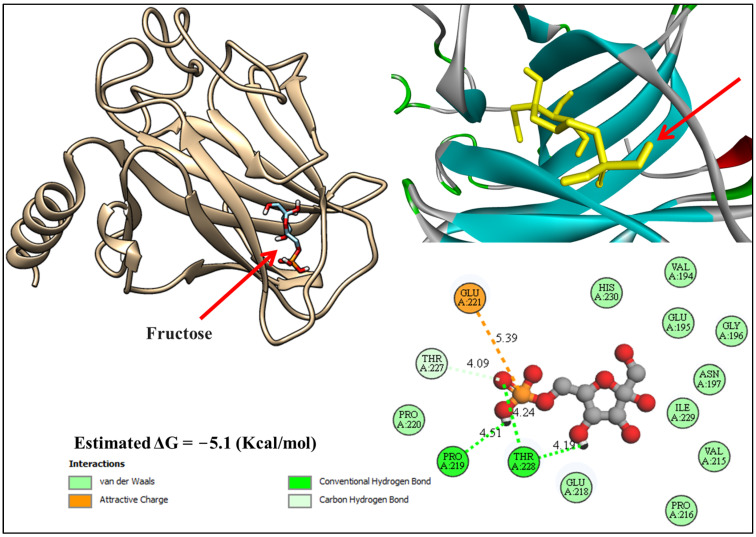
Docking results of fructose with cellular tumor antigen p53 (PDB: 3EXL).

The molecular docking analysis of beta-D-fructose with cellular tumor antigen p53, which had a docking energy level of −5.1 kcal/mol, revealed different bonds, including conventional hydrogen bonds with Proline 219 (4.51 Å) and Threonine 228 (4.24 Å and 4.19 Å), carbon hydrogen bonds with Threonine 227 (4.09 Å), and attractive interactions between fructose and glutamic acid 221 (5.39 Å). These annotated interactions underline the molecular forces stabilizing the binding of fructose within the active site (Figure 3).

**Figure 4:**
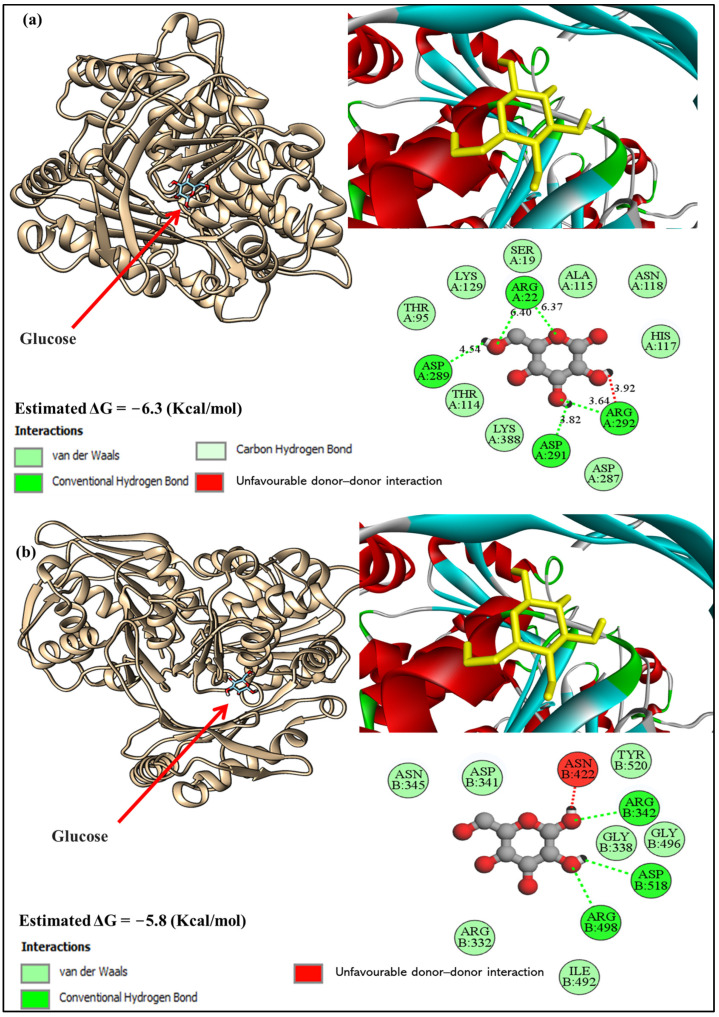
Docking results of glucose with glucokinase (**a**) chain A and chain (**b**) (PDB: 1VKL).

The molecular docking analysis of glucose with glucokinase chain A revealed a docking energy level of −6.3 kcal/mol and the involvement of a network of hydrogen bonds and van der Waals forces that stabilized the molecule within the binding pocket. Among the most significant hydrogen bond interactions, Arginine 22 formed a bond with glucose at approximately 6.40 Å. Another critical hydrogen bond was observed with Arginine 292, which interacted at 3.82 Å. Additionally, Aspartate 289 formed a hydrogen bond with glucose at a bond length of 4.54 Å, and Aspartate 291 contributed with a bond length of 3.64 Å. Residues such as Lysine 129, Alanine 115, and Asparagine 118 formed non-covalent interactions, and Threonine 95 and Threonine 114 also participated in van der Waals forces. Other notable residues included Serine 19 and Histidine 117, which were positioned to support the binding through weaker interactions. Lysine 388 and Aspartate 287 also contributed to the binding through van der Waals forces (Figure 4a).

In Figure 4b, the glucose molecule interacted with the residues of glucokinase chain B with a docking energy level of −5.8 kcal/mol thorough variety of specific interactions, including hydrogen bonds and van der Waals forces. The 2D model revealed that key residues involved in hydrogen bonding were Arginine 342, with a bond length of 6.40 Å, and Arginine 498, which exhibited a strong hydrogen bond at 3.82 Å. Additionally, Aspartate 518 played a crucial role by forming a hydrogen bond with glucose at a bond length of 4.54 Å, while Glycine 496 contributed through another hydrogen bond at 3.64 Å. The van der Waals interactions further reinforced the binding and involved residues such as Asparagine 422, Isoleucine 492, and Tyrosine 520, which were positioned close to glucose, allowing for non-covalent stabilization. Other residues, including Glycine 338, Aspartate 341, and Glycine 496, interacted through weaker van der Waals forces, ensuring the molecule remained properly oriented within the pocket.

#### 3.2.1. Protective Effect of Honey and *Nigella sativa* Oil for Colitis Alleviation

**Figure 5:**
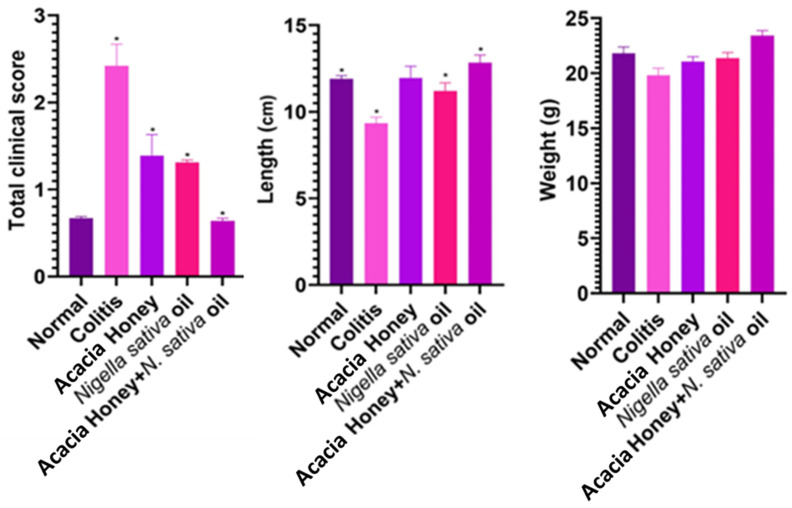
Protective effect of acacia honey, *Nigella sativa* oil, and acacia honey + *Nigella sativa* oil on total clinical score, colon length, and weight loss after colitis. Values expressed are mean ± SE. * *p* < 0.05.

TCSs in the colitis group were significantly higher (2.41 ± 0.25) (*p* < 0.05) than in the normal (control) group (0.66 ± 0.00.) All the treatments significantly (*p* < 0.05) reduced TCSs as compared to the colitis group without honey and NSO administration. The administration of NSO (11.20 ± 0.47) or honey + NSO (9.32 ± 0.34) significantly reduced (*p* < 0.05) the colon length when compared with the colitis group (12.82 ± 0.44). On the other hand, the honey group experienced no significant change (11.95 ± 0.67) (*p* > 0.05) in colon length. No significant differences were observed in animal body weight in any of the treated groups (*p* > 0.05). (Figure 5, Supplementary Table S1)

#### 3.2.2. Oxidative Stress and Enzymatic Markers

TBARS is a product of lipid peroxidation and constitutes a biomarker for oxidative stress. Compared to the control group (*p* < 0.05) (0.28 ± 0.00), TBARS levels increased in the colitis group (0.40 ± 0.18). TBARS activity decreased significantly (*p* < 0.05) in the NSO and honey + NSO groups (0.29 ± 0.02 and 0.15 ± 0.00, respectively) as compared to the colitis group. No significant antioxidant effect was found in group treated with honey (0.34 ± 0.14) (*p* > 0.05). Catalase activity in the colitis group significantly (*p* < 0.05) decreased (0.97 ± 0.00) compared to the control group (2.72 ± 0.00). Among the honey- and NSO-treated groups, reduced CAT activity was found. However, in contrast, there was no significant increase (*p* > 0.05) (2.13 ± 0.05) in catalase activity in the honey + NSO group as compared to groups treated with honey and NSO separately.

**Figure 6:**
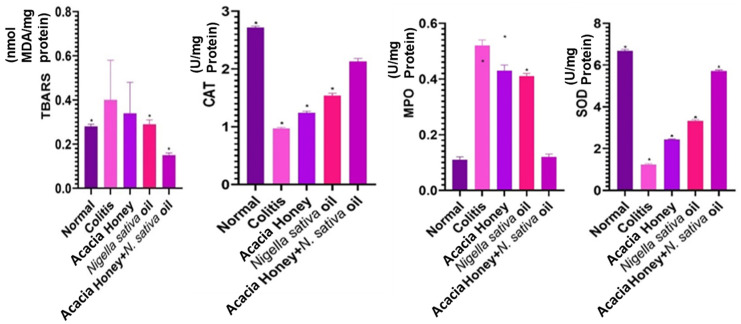
Oxidative stress and enzymatic activity markers of acacia honey, *Nigella sativa* oil, and acacia honey + *Nigella sativa* oil in the colon. Values expressed are mean ± SE. * *p* < 0.05.

There was a significant increase (0.52 ± 0.00) (*p* < 0.05) in MPO activity in the colitis group compared to the control group. Treatment with honey and NSO significantly reduced MPO activity (0.43 ± 0.00 and 0.41 ± 0.00) (*p* < 0.05). Treatment with honey + NSO reduced MPO activity (0.12 ± 0.00) (*p* > 0.05). SOD was comparatively lower (1.23 ± 0.00) (*p* < 0.05) in the colitis group vs. the normal group, whereas treatment with either honey or NSO alone significantly improved SOD activity (2.43 ± 0.03 vs. 3.32 ± 0.00) (*p* < 0.05). Use of the combination honey + NSO treatment showed a significant increase in the colitis-mediated decrease in SOD level (5.72 ± 0.02) (*p* < 0.05), reaching that of the control level (Figure 6, Supplementary Table S2).

#### 3.2.3. Histopathological Study

**Figure 7:**
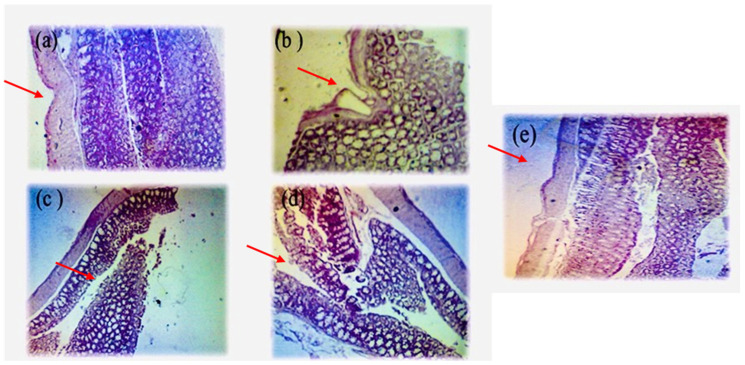
Histopathology of colon. Control group (**a**), colitis group (**b**), acacia honey group (**c**), *Nigella sativa* oil group (**d**), acacia honey + NSO group (**e**). Magnification ×400.

A histological examination of colonic specimen based on the folding of mucosa, extent of inflammation, and depth of inflammatory cell infiltration was carried out. In the NC group, colons showed normal morphology with regular folding of mucosa and no ulceration. (Figure 7a) No signs of regeneration were seen in the untreated group.

In comparison to the control group, the untreated colitis group showed deterioration of the crypt architecture, localized lesions, and epithelial alterations with incomplete mucosa and inflammatory changes (Figure 7b). The group treated with honey exhibited mild inflammatory changes, along with healing and restoration in the mucosal layer (Figure 7c). In the present study, the combination of honey + NSO ameliorated the histological structure. The administration of oil demonstrated a stronger protective effect and lessened the intensity and breadth of colitis. It was found that the use of honey + NSO reduces colonic inflammation with near-complete mucosal regeneration compared to the colitis group (Figure 7e).

## 4. Discussion

The findings from this study underscore the significant therapeutic potential of NSO and AH, individually and in combination, in mitigating oxidative stress, regulating enzymatic markers, and ameliorating colonic damage in colitis-induced models. The results validate their traditional use in gastrointestinal disorders and provide a scientific foundation for their combined efficacy in addressing oxidative and inflammatory pathologies.

Fructose, a major sugar in AH, is involved in critical metabolic pathways [37], interacting with proteins such as: Hexokinases (HK1, HK2, and HK3) [38], which catalyze the phosphorylation of glucose and fructose and are essential for glycolysis [39]. Enhancing glycolysis can provide energy to repair colonic epithelial cells during inflammation [40]; Hexokinase Domain Containing 1 (HKDC1), which regulates glucose homeostasis and is associated with reduced oxidative stress [41]; Platelet and Liver Phosphofructokinase (PFKP, PFKL), which controls the rate-limiting step in glycolysis, promoting energy availability for tissue regeneration [42]; 6-Phosphofructo-2-Kinase/Fructose-2,6-Bisphosphatase (PFKFB). which regulates fructose-2,6-bisphosphate levels, a potent activator of glycolysis [43]. Increased glycolysis may help reduce oxidative damage in inflamed tissues [44]; and TP53-Induced Glycolysis and Apoptosis Regulator (TIGAR), which modulates the balance between glycolysis and antioxidant response by reducing reactive oxygen species (ROS) [45]. The interactions between ligand and proteins suggest that beta-D-fructose has the potential to restore energy metabolism and mitigate oxidative stress, both of which are crucial for colonic healing. Ligand–protein interactions represent positive regulatory interactions, highlighting fructose’s role in enhancing metabolic pathways, while protein–protein interactions indicate synergistic roles.

As thymoquinone interacts with proteins involved in inflammation and apoptosis. Polo-Like Kinase 1 (PLK1) regulates cell division and prevents uncontrolled cell death, promoting colonic epithelial repair [46]. Phosphatase and Tensin Homolog (PTEN) suppresses pro-inflammatory signaling by inhibiting the PI3K/AKT pathway, reducing immune overactivation [47]. Checkpoint Kinase 1 (CHEK1) ensures DNA repair and cell cycle progression under stress conditions [48]. Caspase 3 (CASP3) induces controlled apoptosis, facilitating the removal of damaged epithelial cells. COX-1/COX-2 (PTGS1/PTGS2) regulates prostaglandin synthesis [49]. Thymoquinone selectively inhibits COX-2, reducing inflammation without disrupting protective COX-1 functions [50]. Heme Oxygenase 1 (HMOX1) enhances antioxidant defense, protecting colon tissues from oxidative damage [51]. Nitric Oxide Synthase 2 (NOS2) modulates nitric oxide production, balancing its protective and pro-inflammatory effects [52]. Spermidine/Spermine N1-Acetyltransferase 1 (SAT1) regulates polyamine metabolism, contributing to anti-inflammatory effects [53]. Tumor Protein 53 (TP53) facilitates DNA repair and apoptosis in response to cellular stress [54].

Molecular docking technology has become an indispensable approach for elucidating the molecular mechanisms underlying the interactions between bioactive compounds and their target proteins. In the therapeutic context of colitis, the identification and validation of naturally derived compounds, such as beta-D-fructose from *AH* and thymoquinone from NSO, present a promising avenue for the development of novel anti-inflammatory therapies. Both compounds are well-recognized for their anti-inflammatory and antioxidant properties, making them suitable candidates for addressing the pathophysiological complexities of colitis.

The molecular docking analysis of glucose with glucokinase chains A and B provided insightful information about the binding interactions and stabilization mechanisms within the enzyme’s binding pocket. The docking energy levels of −6.3 kcal/mol for chain A and −5.8 kcal/mol for chain B suggest strong binding affinities, highlighting the specificity of the glucose–glucokinase interaction. In chain A, hydrogen bonding played a critical role in stabilizing the glucose molecule. Arginine 22, with a bond length of approximately 6.40 Å, and Arginine 292, with a stronger hydrogen bond at a length of 3.82 Å, were key contributors [55]. Additionally, Aspartate 289 and Aspartate 291 formed hydrogen bonds with glucose at bond lengths of 4.54 Å and 3.64 Å, respectively. These interactions underscore the importance of polar residues in maintaining structural stability and enhancing the binding affinity of glucose [56]. Non-covalent interactions, such as those formed by Lysine 129, Alanine 115, Asparagine 118, Threonine 95, and Threonine 114, further reinforced the binding, with van der Waals forces playing a complementary stabilizing role [57]. Residues like Serine 19 and Histidine 117 provided additional support through weaker interactions, while Lysine 388 and Aspartate 287 contributed significantly to the overall stability of the complex.

For chain B, the interactions followed a similar trend, with key residues participating in hydrogen bonding and van der Waals forces. Arginine 342 and Arginine 498 formed crucial hydrogen bonds at lengths of 6.40 Å and 3.82 Å, respectively. Aspartate 518 and Glycine 496 also established hydrogen bonds with lengths of 4.54 Å and 3.64 Å, emphasizing the role of both charged and neutral residues in the binding process. Van der Waals interactions were prominent, with Asparagine 422, Isoleucine 492, and Tyrosine 520 being key contributors to the stabilization of glucose within the binding pocket. The presence of residues like Glycine 338 and Aspartate 341, which interacted through weaker van der Waals forces, highlighted the cooperative nature of these non-covalent interactions [58] in maintaining the proper orientation of glucose within chain B.

The comparison of docking energies and residue interactions between chains A and B indicates subtle differences in binding affinity and stabilization mechanisms. The slightly higher docking energy in chain A (−6.3 kcal/mol) compared to chain B (−5.8 kcal/mol) suggests a marginally stronger interaction in chain A, potentially due to the specific arrangement of key residues and their hydrogen bonding patterns. However, both chains exhibit robust interaction networks, reflecting the efficiency and specificity of glucokinase in binding glucose.

These findings are consistent with the physiological role of glucokinase as a sensor of glucose levels in the body, emphasizing the importance of strong and specific interactions for effective enzymatic function [59]. The detailed mapping of hydrogen bonds and van der Waals forces in this study provides valuable insights into the structural dynamics of glucokinase–glucose interactions, which may be useful in the design of modulators for therapeutic purposes.

This study integrated in silico molecular docking analyses to predict the binding affinities and interaction profiles of these bioactive compounds with key proteins involved in colitis pathogenesis. Furthermore, the computational findings were corroborated through experimental validation in an albino mice model to assess their therapeutic efficacy and underlying mechanisms of action.

To understand the etiology of colitis, we used an albino mice model. The elevated TCSs in the colitis group reflect the hallmark inflammatory response of the disease, and their significant reduction following treatment supports the anti-inflammatory potential of the interventions [60]. The reduction in colon length observed in the colitis group further corroborates the pathological changes induced by colitis, as inflammation and tissue damage typically result in fibrosis and contraction of the colon [61]. The ability of NSO and AH + NSO to significantly restore colon length indicates their role in attenuating inflammation and promoting tissue repair. The lack of significant improvement in colon length with AH alone suggests that its therapeutic effects may not be sufficient on their own to counteract the structural impacts of colitis, emphasizing the enhanced efficacy of NSO, especially in combination therapies. The absence of significant weight changes among treated groups demonstrates that the treatments were well-tolerated, which is crucial for their potential therapeutic application. This finding supports the safety profile of NSO and its combinations, further validating their use in colitis management. A study reported by Rezaei et al. [31] revealed that honey reduces total clinical scores in experimentally inducing colitis, as was found in this study. The current study’s results are in agreement the study conducted by Prakash et al. [62] in that they revealed an increased level of TBARS as result of lipid peroxidation in experimentally induced colitis, while treatment with honey and NSO significantly reduced TBARS levels. MPO is an essential constituent of neutrophils. Briga et al. [63] observed that NSO consumption plays an important role in the suppression of free radicals as compared to pure honey. Reactive oxygen species are released when TNF alpha levels increase, and this triggers the immune system to attack and kill malignant cells.

According to Rana et al. [64], a decrease in antioxidant activities might be due to the overwhelming effects of free radicals. In the mammalian colon, antioxidant enzymes such as SOD, MPO, CAT, and GPX are present in small amounts [65]. These antioxidant enzymes are free radical scavengers and protect tissues and cells against toxic free radicals. The results of this work demonstrated that treatment with honey, NSO, or a combination of both showed improvements in TBARS, MPO, SOD and CAT activity that were similar to those found by Prakash et al. [62]. Previous results have suggested that NSO can adjust intestinal inflammation in IBD through the mechanisms of immunity regulation and intestinal flora [66].

Histological assessment showed that the colitis group presented the histological features of ulcerative colitis. A study performed by Grisham et al. [67] revealed that TNBS-induced colitis causes the infiltration of submucosal leukocytes, which contributes noticeably to tissue damage (TNBS, 2,4,6-trinitrobenzenesulfonic acid, is a chemical compound commonly used to induce experimental colitis in animal models, CAS no. 2508-19-2). Myeloperoxidase present in neutrophils catalyzes the formation of cytotoxic oxidants such as hypochlorous acid. Leukocyte infiltration indicates tissue injury and mucosal disfunction [68]. In the present research, a significant reduction in tissue injury was observed at a microscopic level. NSO plays a significant role in inflammatory diseases [69]. In an earlier study, Bilsel et al. [70] showed that honey can be used against colitis. Our study showed that the combination of two natural compounds ameliorated the histological structure.

## 5. Conclusions

The study highlights the therapeutic efficacy of NSO and AH in mitigating oxidative stress, modulating enzymatic markers, and promoting colonic healing in colitis-induced models. Molecular docking and protein–protein interaction analysis reveal the role of key compounds, such as thymoquinone and fructose, in regulating metabolic and inflammatory pathways, including glycolysis, apoptosis, and oxidative stress responses. This dual approach of biochemical and molecular insights underscores their potential as complementary agents in managing gastrointestinal inflammation and oxidative damage.

## Data Availability

The relevant required datasets can be obtained from the corresponding author upon special request.

## Supplementary Materials

The following supporting information can be downloaded at: https://www.mdpi.com/article/10.3390/biology13121035/s1, Table S1: Protective effect of honey, NSO, and honey + NSO on total clinical score, colon length, and weight loss after colitis; Table S2: Protective effect of honey, NSO, and honey + NSO on biochemical parameters.

## Abbreviations

Ulcerative colitis (UC), *Nigella sativa oil* (NSO), Prostaglandin G/H synthase 2 (Ptgs2), inflammatory bowel disease (IBD), AH group (AH), negative control (NC), positive control (PC), total clinical scores (TCS), thiobarbituric acid reactive substances (TBARS), superoxide dismutase (SOD), catalase (CAT), myeloperoxidase (MPO), phosphate-buffered saline (PBS), trichloroacetic acid (TCA), nitroblue tetrazolium (NBT), hydrogen peroxide (H_2_O_2_), Statistical Package for the Social Sciences (SPSS), standard error of the mean (SEM), analysis of variance (ANOVA), Hexokinases (HK1, HK2, and HK3), Hexokinase Domain Containing 1 (HKDC1), Platelet and Liver Phosphofructokinase (PFKP, PFKL), 6-Phosphofructo-2-Kinase/Fructose-2,6-Bisphosphatase (PFKFB), TP53-Induced Glycolysis and Apoptosis Regulator (TIGAR), reactive oxygen species (ROS), Polo-Like Kinase 1 (PLK1), Phosphatase and Tensin Homolog (PTEN), Checkpoint Kinase 1 (CHEK1), Caspase 3 (CASP3), Heme Oxygenase 1 (HMOX1), Nitric Oxide Synthase 2 (NOS2), Spermidine/Spermine N1-Acetyltransferase 1 (SAT1), Tumor Protein 53 (TP53).

## References

[1] Xu J., Liu T., Shao Y., Liu Q., Zhang Z., Yuan Y., Zhang S., Wang Y., Sun L., Zhou S. (2024). Phage cocktail inhibits inflammation and protects the integrity of the intestinal barrier in dextran sulfate sodium-induced colitis mice model. Microb. Pathog..

[2] Cannarozzi A.L., Latiano A., Massimino L., Bossa F., Giuliani F., Riva M., Ungaro F., Guerra M., Di Brina A.L., Biscaglia G. (2024). Inflammatory bowel disease genomics, transcriptomics, proteomics and metagenomics meet artificial intelligence. United Eur. Gastroenterol. J..

[3] Diez-Martin E., Hernandez-Suarez L., Muñoz-Villafranca C., Martin-Souto L., Astigarraga E., Ramirez-Garcia A., Barreda-Gómez G. (2024). Inflammatory Bowel Disease: A Comprehensive Analysis of Molecular Bases, Predictive Biomarkers, Diagnostic Methods, and Therapeutic Options. Int. J. Mol. Sci..

[4] Liu J., Di B., Xu L.L. (2023). Recent advances in the treatment of IBD: Targets, mechanisms and related therapies. Cytokine Growth Factor Rev..

[5] Najmi A., Javed S.A., Al Bratty M., Alhazmi H.A. (2022). Modern approaches in the discovery and development of plant-based natural products and their analogues as potential therapeutic agents. Molecules.

[6] Qayyum S., Sultana A., Heyat MB B., Rahman K., Akhtar F., Haq A.U., Alkhamis B.A., Alqahtani M.A., Gahtani R.M. (2023). Therapeutic efficacy of a formulation prepared with *Linum usitatissimum* L., *Plantago ovata* Forssk., and honey on uncomplicated pelvic inflammatory disease analyzed with machine learning techniques. Pharmaceutics.

[7] Kumar R., Kumar S., Kanwar S.S. (2024). Herbal-Infused Honey vis-à-vis Human Health. Biomedical Perspectives of Herbal Honey.

[8] Abbas M., Gururani M.A., Ali A., Bajwa S., Hassan R., Batool S.W., Imam M., Wei D. (2024). Antimicrobial Properties and Therapeutic Potential of Bioactive Compounds in *Nigella sativa*: A Review. Molecules.

[9] Iftikhar A., Nausheen R., Mukhtar I., Iqbal R.K., Raza A., Yasin A., Anwar H. (2023). The regenerative potential of honey: A comprehensive literature review. J. Apic. Res..

[10] Hamadou W.S., Bouali N., Badraoui R., Hadj Lajimi R., Hamdi A., Alreshidi M., Patel M., Adnan M., Siddiqui A.J., Noumi E. (2022). Chemical composition and the anticancer, antimicrobial, and antioxidant properties of AH from the Hail region: The in vitro and in silico investigation. Evid.-Based Complement. Altern. Med..

[11] Sun W., Shahrajabian M.H. (2023). Therapeutic potential of phenolic compounds in medicinal plants—Natural health products for human health. Molecules.

[12] Chen S., Wang X., Cheng Y., Gao H., Chen X. (2023). A review of classification, biosynthesis, biological activities and potential applications of flavonoids. Molecules.

[13] Caban M., Lewandowska U. (2022). Polyphenols and the potential mechanisms of their therapeutic benefits against inflammatory bowel diseases. J. Funct. Foods.

[14] Imran M., Khan S.A., Abida, Alshammari M.K., Alkhaldi S.M., Alshammari F.N., Kamal M., Alam O., Asdaq S.M.B., Alzahrani A.K. (2022). *Nigella sativa* L. and COVID-19: A glance at the anti-COVID-19 chemical constituents 2022, clinical trials, inventions, and patent literature. Molecules.

[15] Kohandel Z., Farkhondeh T., Aschner M., Samarghandian S. (2021). Anti-inflammatory effects of thymoquinone and its protective effects against several diseases. Biomed. Pharmacother..

[16] Venkataraman B., Almarzooqi S., Raj V., Alhassani A.T., Alhassani A.S., Ahmed K.J., Subramanian V.S., Ojha S.K., Attoub S., Adrian T.E. (2021). Thymoquinone, a dietary bioactive compound, exerts anti-inflammatory effects in colitis by stimulating expression of the colonic epithelial PPAR-γ transcription factor. Nutrients.

[17] Gholamnezhad Z., Keyhanmanesh R., Boskabady M.H. (2015). Anti-inflammatory, antioxidant, and immunomodulatory aspects of *Nigella sativa* for its preventive and bronchodilatory effects on obstructive respiratory diseases: A review of basic and clinical evidence. J. Funct. Foods.

[18] Agu P.C., Afiukwa C.A., Orji O.U., Ezeh E.M., Ofoke I.H., Ogbu C.O., Ugwuja E.I., Aja P.M. (2023). Molecular docking as a tool for the discovery of molecular targets of nutraceuticals in diseases management. Sci. Rep..

[19] Akash S., Shanto SH I., Islam M.R., Bayil I., Afolabi S.O., Guendouzi A., Abdellattif M.H., Zaki M.E. (2024). Discovery of novel MLK4 inhibitors against colorectal cancer through computational approaches. Comput. Biol. Med..

[20] Sadybekov A.V., Katritch V. (2023). Computational approaches streamlining drug discovery. Nature.

[21] Vidal-Limon A., Aguilar-Toalá J.E., Liceaga A.M. (2022). Integration of molecular docking analysis and molecular dynamics simulations for studying food proteins and bioactive peptides. J. Agric. Food Chem..

[22] Druzhilovskiy D.S., Rudik A.V., Filimonov D.A., Gloriozova T.A., Lagunin A.A., Dmitriev A.V., Pogodin P.V., Dubovskaya V.I., Ivanov S.M., Tarasova O.A. (2017). Computational platform Way2Drug: From the prediction of biological activity to drug repurposing. Russ. Chem. Bull..

[23] Nigatu T.A., Afework M., Urga K., Ergete W., Makonnen E. (2017). Toxicological investigation of acute and chronic treatment with Gnidia stenophylla Gilg root extract on some blood parameters and histopathology of spleen, liver and kidney in mice. BMC Res. Notes.

[24] Olovo C.V., Wiredu Ocansey D.K., Ji Y., Huang X., Xu M. (2024). Bacterial membrane vesicles in the pathogenesis and treatment of inflammatory bowel disease. Gut Microbes.

[25] Fatima Shad K., Soubra W., Cordato D.J. (2021). The role of thymoquinone, a major constituent of *Nigella sativa*, in the treatment of inflammatory and infectious diseases. Clin. Exp. Pharmacol. Physiol..

[26] Ansary J., Giampieri F., Forbes-Hernandez T.Y., Regolo L., Quinzi D., Villar S.G., Villena E.G., Pifarre K.T., Alvarez-Suarez J.M., Battino M. (2021). Nutritional value and preventive role of *Nigella sativa* L. and its main component thymoquinone in cancer: An evidenced-based review of preclinical and clinical studies. Molecules.

[27] Valverde S., Ares A.M., Elmore J.S., Bernal J. (2022). Recent trends in the analysis of honey constituents. Food Chem..

[28] Hossain M.M., Nath Barman D., Rahman M.A., Khandker S.S. (2023). Carbohydrates in Honey. Honey Compos. Health Benefits.

[29] Jabbar M., Baboo I., Majeed H., Farooq Z., Palangi V. (2024). Characterization and antibacterial application of peppermint essential oil nanoemulsions in broiler. Poult. Sci..

[30] Jabbar M., Baboo I., Majeed H., Farooq Z., Palangi V., Lackner M. (2024). Preparation and Characterization of Cumin Essential Oil Nanoemulsion (CEONE) as an Antibacterial Agent and Growth Promoter in Broilers: A Study on Efficacy, Safety, and Health Impact. Animals.

[31] Rezaei N., Eftekhari M.H., Tanideh N., Mokhtari M., Bagheri Z. (2019). Comparison of antioxidant and anti-inflammatory effects of honey and *spirulina platensis* with sulfasalazine and mesalazine on acetic acid-induced ulcerative colitis in rats. Galen Med. J..

[32] Zhu Q., Han Y., Wang X., Jia R., Zhang J., Liu M., Zhang W. (2023). Hypoxia exacerbates intestinal injury and inflammatory response mediated by myeloperoxidase during *Salmonella typhimurium* infection in mice. Gut Pathog..

[33] Ghani M.A., Barril C., Bedgood D.R., Prenzler P.D. (2017). Measurement of antioxidant activity with the thiobarbituric acid reactive substances assay. Food Chem..

[34] Weydert C.J., Cullen J.J. (2010). Measurement of superoxide dismutase, catalase and glutathione peroxidase in cultured cells and tissue. Nat. Protoc..

[35] López-Mejía A., Ortega-Pérez L.G., Magaña-Rodríguez O.R., Ayala-Ruiz L.A., Piñón-Simental J.S., Hernández D.G., Rios-Chavez P. (2021). Protective effect of Callistemon citrinus on oxidative stress in rats with 1, 2-dimethylhydrazine-induced colon cancer. Biomed. Pharmacother..

[36] Marotti V., Xu Y., Michalowski C.B., Zhang W., Domingues I., Ameraoui H., Moreels T.G., Baatsen P., Van Hul M., Muccioli G.G. (2024). A nanoparticle platform for combined mucosal healing and immunomodulation in inflammatory bowel disease treatment. Bioact. Mater..

[37] Chen S., Wu F., Yang C., Zhao C., Cheng N., Cao W., Zhao H. (2022). Alternative to sugar, honey does not provoke insulin resistance in rats based on lipid profiles, inflammation, and IRS/PI3K/AKT signaling pathways modulation. J. Agric. Food Chem..

[38] Nazemi-Rafie J., Fatehi F., Hasrak S. (2023). A comparative transcriptome analysis of the head of 1 and 9 days old worker honeybees (*Apis mellifera*). Bull. Entomol. Res..

[39] Qi X., Tester R.F. (2019). Fructose, galactose and glucose–In health and disease. Clin. Nutr. ESPEN.

[40] Ahmed S., Sulaiman S.A., Baig A.A., Ibrahim M., Liaqat S., Fatima S., Jabeen S., Shamim N., Othman N.H. (2018). Honey as a potential natural antioxidant medicine: An insight into its molecular mechanisms of action. Oxidative Med. Cell. Longev..

[41] Khan M.W., Priyadarshini M., Cordoba-Chacon J., Becker T.C., Layden B.T. (2019). Hepatic hexokinase domain containing 1 (HKDC1) improves whole body glucose tolerance and insulin sensitivity in pregnant mice. Biochim. Et Biophys. Acta (BBA)-Mol. Basis Dis..

[42] Zuo J., Tang J., Lu M., Zhou Z., Li Y., Tian H., Liu E., Gao B., Liu T., Shao P. (2021). Glycolysis rate-limiting enzymes: Novel potential regulators of rheumatoid arthritis pathogenesis. Front. Immunol..

[43] Xiao J., Zhou Y., Xie Y., Li T., Su X., He J., Jiang Y., Zhu H., Qu H. (2024). ATP homeostasis and signalling in plants. Plant Commun..

[44] Pająk B., Zieliński R., Priebe W. (2024). The Impact of Glycolysis and Its Inhibitors on the Immune Response to Inflammation and Autoimmunity. Molecules.

[45] Li L., Xu T., Qi X. (2024). Balanced regulation of ROS production and inflammasome activation in preventing early development of colorectal cancer. Immunol. Rev..

[46] Zhang Z., Xing X., Guan P., Song S., You G., Xia C., Liu T. (2021). Recent progress in agents targeting polo-like kinases: Promising therapeutic strategies. Eur. J. Med. Chem..

[47] Yu J., Huang J., Xia T., Li M., Li R. (2024). Molecular mechanisms of hepatic lipid metabolism disorders: Focus on mitochondrial quality control. Portal Hypertens. Cirrhosis.

[48] Patil M., Pabla N., Dong Z. (2013). Checkpoint kinase 1 in DNA damage response and cell cycle regulation. Cell. Mol. Life Sci..

[49] Asadi M., Taghizadeh S., Kaviani E., Vakili O., Taheri-Anganeh M., Tahamtan M., Savardashtaki A. (2022). Caspase-3: Structure, function, and biotechnological aspects. Biotechnol. Appl. Biochem..

[50] El Mezayen R., El Gazzar M., Nicolls M.R., Marecki J.C., Dreskin S.C., Nomiyama H. (2006). Effect of thymoquinone on cyclooxygenase expression and prostaglandin production in a mouse model of allergic airway inflammation. Immunol. Lett..

[51] Puentes-Pardo J.D., Moreno-SanJuan S., Carazo Á., León J. (2020). Heme oxygenase-1 in gastrointestinal tract health and disease. Antioxidants.

[52] Lan J., Wang J., Wang S., Wang J., Huang S., Wang Y., Ma Y. (2024). The Activation of GABAAR Alleviated Cerebral Ischemic Injury via the Suppression of Oxidative Stress, Autophagy, and Apoptosis Pathways. Antioxidants.

[53] Yuan F., Zhang L., Cao Y., Gao W., Zhao C., Fang Y., Zahedi K., Soleimani M., Lu X., Fang Z. (2018). Spermidine/spermine N1-acetyltransferase-mediated polyamine catabolism regulates beige adipocyte biogenesis. Metabolism.

[54] Shahbazi J., Lock R., Liu T. (2013). Tumor protein 53-induced nuclear protein 1 enhances p53 function and represses tumorigenesis. Front. Genet..

[55] Dong J., Davis A.P. (2021). Molecular recognition mediated by hydrogen bonding in aqueous media. Angew. Chem..

[56] Guo W., Mehrparvar S., Hou W., Pan J., Aghbashlo M., Tabatabaei M., Rajaei A. (2024). Unveiling the impact of high-pressure processing on anthocyanin-protein/polysaccharide interactions: A comprehensive review. Int. J. Biol. Macromol..

[57] Yan Z., Liu X., Ding B., Yu J., Si Y. (2023). Interfacial engineered superelastic metal-organic framework aerogels with van-der-Waals barrier channels for nerve agents decomposition. Nat. Commun..

[58] Samanta P.N., Majumdar D., Roszak S., Leszczynski J. (2022). First-Principles Modeling of Non-covalent Interactions in Molecular Systems and Extended Materials. Practical Aspects of Computational Chemistry V.

[59] Abu Aqel Y., Alnesf A., Aigha I.I., Islam Z., Kolatkar P.R., Teo A., Abdelalim E.M. (2024). Glucokinase (GCK) in diabetes: From molecular mechanisms to disease pathogenesis. Cell. Mol. Biol. Lett..

[60] Ren J., Yue B., Wang H., Zhang B., Luo X., Yu Z., Zhang J., Ren Y., Mani S., Wang Z. (2021). Acacetin ameliorates experimental colitis in mice via inhibiting macrophage inflammatory response and regulating the composition of gut microbiota. Front. Physiol..

[61] Crafa P., Diaz-Cano S.J. (2022). Changes in colonic structure and mucosal inflammation. Colonic Diverticular Disease.

[62] Prakash A., Medhi B., Avti P.K., Saikia U.N., Pandhi P., Khanduja K.L. (2008). Effect of different doses of Manuka honey in experimentally induced inflammatory bowel disease in rats. Phytother. Res. Int. J. Devoted Pharmacol. Toxicol. Eval. Nat. Prod. Deriv..

[63] Briga M., Odobasic A., Sestan I., Begic S. (2019). Black seed oil as an additive to honey. Eur. Food Res. Technol..

[64] Rana S.V., Sharma S., Kaur J., Prasad K.K., Sinha S.K., Kochhar R., Morya R.K. (2014). Relationship of cytokines, oxidative stress and GI motility with bacterial overgrowth in ulcerative colitis patients. J. Crohn’s Colitis.

[65] Verspaget H.W., Mulder TP J., Van der Sluys Veer A., Pea A.S., Lamers CB H.W. (1991). Reactive oxygen metabolites and colitis; a disturbed balance between damage and protection: A selective review. Scand. J. Gastroenterol..

[66] Hu X., Xu N., Yang X., Hu X., Zheng Y., Zhang Q. (2020). *Nigella* ameliorates inflammation and intestinal flora imbalance in DSS induced colitis mice. Appl. Microbiol. Biotechnol. Express.

[67] Grisham M.B., Volkmer C., Tso P., Yamada T. (1991). Metabolism of trinitrobenzene sulfonic acid by the rat colon produces reactive oxygen species. Gastroenterology.

[68] Islam M.S., Murata T., Fujisawa M., Nagasaka R., Ushio H., Bari A.M., Ozaki H. (2008). Anti-inflammatory effects of phytosteryl ferulates in colitis induced by dextran sulphate sodium in mice. Br. J. Pharmacol..

[69] Rashwan H.K., Mahgoub S., Abuelezz N.Z., Amin H.K. (2023). Black cumin seed (*Nigella sativa*) in inflammatory disorders: Therapeutic potential and promising molecular mechanisms. Drugs Drug Candidates.

[70] Bilsel Y., Bugra D., Yamaner S., Bulut T., Cevikbas U., Turkoglu U.J.D.S. (2002). Could honey have a place in colitis therapy? Effects of honey, prednisolone, and disulfiram on inflammation, nitric oxide, and free radical formation. Dig. Surg..

