# Supplementary material for: Molecular Docking of Key Compounds from Acacia Honey and Nigella sativa Oil and Experimental Validation for Colitis Treatment in Albino Mice
Source: Biology (Basel). 2024 Dec 11;13(12):1035. doi: 10.3390/biology13121035 (PMC11673436; doi:10.3390/biology13121035)
Supplement: Supplementary file 1 [file biology-13-01035-s001.zip › biology-3335037-supplementary.pdf]

Table S1. Protective effect of honey, NSO and honey + NSO on total clinical score, colon length and weight loss after Colitis. Values expressed are Mean ± SE.*p<0.05

**Morphological parameters**	**Experimental groups**				
	**Control**	**Colitis**	**Honey**	**NSO**	**Honey+ NSO**
Total clinical score (TCS)	0.66 ± 0.00	2.41 ± 0.25*	1.38 ± 0.24*	1.30 ± 0.02*	0.63 ± 0.02*
Colon length (cm)	11.90 ± 0.18*	12.82±0.44*	11.95 ± 0.67	11.20 ± 0.47*	9.32 ± 0.34*
Weight loss (g)	21.80 ± 0.57	19.80 ±0.64	21.05 ± 0.42	21.35 ± 0.51	23.40± 0.47

NSO: ***Nigella sativa*** **oil**

Table S2. Protective effect of honey, NSO and honey + NSO on Biochemical parameters. Values expressed are Mean ± SE. *p<0.05

**Biochemical parameters**	**Experimental groups**				
	**Control**	**Colitis**	**Honey**	**NSO**	**Honey+ NSO**
TBARS (nmol MDA/mg protein)	0.28 ± 0.00*	0.40± 0.18	0.34 ± 0.14	0.29 ± 0.02*	0.15 ± 0.00*
CAT (U/mg Protein)	2.72 ± 0.00*	0.97 ± 0.00*	1.24 ± 0.00*	1.54 ± 0.00*	2.13± 0.05
MPO (U/mg Protein)	0.11 ± 0.00	0.52 ± 0.00*	0.43 ± 0.00*	0.41 ± 0.00*	0.12± 0.00
SOD (U/mg Protein)	6.68 ± 0.00*	1.23 ± 0.00*	2.43 ± 0.03*	3.32 ± 0.00*	5.72 ± 0.02*

NSO: ***Nigella sativa*** **oil**
